# Early development of a novel scale to measure adaptation in people diagnosed with inflammatory bowel disease - the A-inflammatory bowel disease

**DOI:** 10.1177/20551029221098550

**Published:** 2022-05-17

**Authors:** Lawrence Matini, James Ogden

**Affiliations:** 1 School of Psychology, 3660 University of Surrey, Guildford, UK

**Keywords:** adaptation, chronic illness, sense making

## Abstract

**Objective:**

To undergo the preliminary development of a new measure of patient adaptation to Inflammatory Bowel Disease (IBD): A-IBD.

**Design:**

Based on a prior conceptualisation of adaptation, a 40-item scale was generated and completed by 304 people diagnosed with IBD.

**Main outcome measures:**

Psychometric analysis of the measure. Association with the Brief Illness Perception Questionnaire (Brief IPQ) and the Inflammatory Bowel Disease Questionnaire (IBDQ).

**Results:**

The 18-item scale consisted of four subscales (patient identity, person identity, acceptance, expectations). Weak to moderate correlations were found between subscales of the A-IBD and the Brief IPQ and IBDQ.

**Conclusion:**

The A-IBD shows potential for assessing adaptation. Further analysis could confirm its usefulness.

## Introduction

Inflammatory Bowel Disease (IBD) is a term that is used mainly to describe two chronic, relapsing-remitting conditions of the gastrointestinal tract, namely Crohn’s Disease (CD) and Ulcerative Colitis (UC). While CD and UC are relatively distinct in their presenting location within the GI tract, they are grouped together under the umbrella of IBD due to their common symptoms, gastrointestinal and systemic complications and their characteristic phases of disease exacerbation and intermittent remission (Kirsner, 1995). During periods of active disease, people diagnosed with either CD or UC frequently report a range of simultaneous symptoms typically involving acute abdominal pain and spasms, diarrhoea, stool incontinence, rectal bleeding, nausea, fever and fatigue (Langmead and Irving, 2008; Norton et al., 2013). Furthermore, fatigue often persists even in times of remission which in turn negatively impacts upon patients’ daily functioning (Czuber-Dochan et al., 2013a; 2013b). People diagnosed with IBD have also described how both incontinence, as well as the fear of it, limited their social, working and personal lives (Dibley and Norton, 2013) and how it has interfered with work and educational performance (Lonnfors et al., 2014). Research has therefore explored the psychological impact of IBD both in terms of the symptoms it produces as well as the wider, global impact of these symptoms.

Research also indicates raised levels of psychological comorbidities in this population. For example, one study found that almost 20% of people diagnosed with IBD have at least one anxiety condition, while just over 10% suffer from a depressive disorder (Tribbick et al., 2015). In turn, these psychological comorbidities have also been found to reduce medication adherence (Gray et al., 2012) which as well as being associated with recurrence of symptoms (Mikocka-Walus et al., 2016), in turn increases the risk of an anxiety disorder up to around 80% (Nahon et al., 2012).

Disease management, which in the absence of any definitive cure remains the main focus for IBD patients, can also generate a range of psychological, social and behavioural problems. For example, corticosteroid treatment for IBD has been implicated in mood changes (Christensen, 2014), compounding any existing issues of anxiety or depression. Further, the commonplace need for surgery results in abdominal scarring, and sometimes stomas and ostomy bags, which can lead to body image dissatisfaction which has been reported by both men and women regardless of improvements in disease activity (Saha et al., 2015). Body image issues are also linked to steroidal treatment which can cause facial swelling and acne and has been associated with a higher rate of depressive symptoms (Reed-Knight et al., 2014). Such consequences of disease management can also impact upon the broader aspects of daily life such as the desire to take part in social activities (McDermott et al., 2015), intimate relationships (Jedel et al., 2015) and going to work or school (Lonnfors et al., 2014). In turn, research indicates that IBD is also linked with poor health-related quality of life (HRQoL) (Floyd et al., 2015), as well decreased general life satisfaction (Janke et al., 2004) even in times of remission, suggesting that these may be a result of merely having the disease (Drossman et al., 1989; Lix et al., 2008).

IBD is therefore all-encompassing for patients, challenging them on a multitude of levels and contributing to psychological, social and behavioural problems which result in poor quality of life. Adaptation itself in health contexts is a term loosely used to describe a variety of psychosocial and behavioural skills and techniques that help patients manage their condition. To date, however, little is known concerning what adaptation means to people diagnosed with IBD or how it can be operationalised and potentially subsequently measured.

Although little has been written about adaptation in people diagnosed with IBD, much chronic illness research exists that has investigated adaptation (and various related concepts including adjustment and acceptance) in patients with a multitude of different health backgrounds. For example, drawing upon Cognitive Adaptation Theory (Taylor, 1983), studies have explored adaptation in those treated for coronary artery disease (Helgeson, 2003), late-stage cancer of varying types (Christianson et al., 2013), non-melanoma skin cancer (Czajkowska et al., 2013) and type 1 diabetes (Helgeson et al., 2014). This approach is framed by three underlying processes: a search for meaning, attempting to regain mastery and self enhancement. In contrast, other studies have drawn upon the Self-Regulation Model (Leventhal et al., 1980), focusing on conditions such as Chronic Fatigue Syndrome, eating disorders and arthritis (Moss-Morris et al., 1996; Marcos et al., 2007; Pimm and Weinman, 1998). From this perspective, adaptation is seen as a product of illness and emotional representations. Both models, however, take a limited approach to the illness experience, and whilst they emphasise sense making they neglect the biopsychosocial nature of the condition. Furthermore, the operationalisation of the notion of adaptation remains unclear.

As a means to capture this broader approach to IBD, Devlen et al. (2014) developed their own conceptual model of the impact of IBD which demonstrates the interconnectedness of patients’ bowel and systemic symptoms not only with the psychological impact of the disease, but also with the impacts of treatment, relationship difficulties, social and leisure activity and work, school and parenting limitations. Although broader in its perspective than the aforementioned models, this model also neglected to operationalise adaptation, leaving it as the product of a number of illness consequences rather than an entity in its own right. Likewise, whilst Voth and Sirois (2009) measured adjustment to IBD, adjustment here was operationalised as a composite of coping efficacy, acceptance and helplessness rather than a distinct construct. Other research in IBD has merely emphasised adaptation as a form of coping (Jones et al., 2006; McCombie et al., 2015; Moskovitz et al., 2000). Therefore, whilst models such as the aforementioned CAT or SRM could be used as a basis to study adaptation in IBD, their focus on sense making neglects the broader impact of this condition and leaves the definition of adaptation unclear. Research specific to IBD may be broader in its perspective, but again either offers an unclear definition of what this construct means or simply equates it with other psychological processes.

The present paper argues that adaptation is core to the experiences of those with IBD. In addition, it argues that in order to be measured effectively this construct requires both clear conceptualisation and operationalisation. This was the focus of a previous qualitative study which explored how people diagnosed with IBD adapted to their illness as a means of aiding the early development of the measure presented in the current paper (Matini and Ogden, 2015). In this study, 20 patients were interviewed concerning their experiences of adapting to life with IBD. The results were analysed using thematic analysis and indicated that adaptation involved the search for a ‘new normal’ with patients striving to find psychological, emotional and behavioural equilibrium between their identity as a patient with IBD and that of being a person existing beyond their illness identity. The results from the study also emphasised the relational processes that occur between the patient and their illness. Accordingly, the self and the disease are better conceptualised as interconnected and in a dynamic dyad with patients moving dynamically in this instance along a spectrum from ‘person identity’ to ‘patient identity’. Moss-Morris’ (2013) working model on ‘adjusting’ to chronic illness does indeed state that if a disease becomes progressive, factors such as acceptance and self-compassion may become more important, touching on the nature of adaptation as an iterative, ongoing process.

In summary, it is clear that the notion of adaptation is central to the experiences of those with IBD. Although more generic models such as the CAT and SRM (Leventhal et al., 1980; Taylor , 1983) provide some insights into this construct, it remains poorly conceptualised and therefore difficult to measure. Therefore, the aim of the present study was to draw upon the findings of Matini and Ogden’s (2015) qualitative study as a means to conceptualise adaptation to IBD as involving the striving for psychological, emotional and behavioural equilibrium which involves a ‘new normal’, reflecting a balance between the identity as a person and that as a patient with a disease. In doing so, the current study aimed to take the first steps towards the development of a new measure of adaptation to IBD which could be used to eventually quantify adaptation in this patient group. This prospective measure was operationalised based upon the prior qualitative data, subjected to an exploratory factor analysis and then assessed in terms of its association with an existing measure of sense making - the Brief Illness Perception Questionnaire (Broadbent et al., 2006) and an existing measure of IBD-related quality of life – the Inflammatory Bowel Disease Questionnaire (Guyatt et al., 1989). In doing so, we sought to determine whether adaptation is indeed a unique construct, as has thus far been argued, and if we can take the first steps towards the development of a reliable and valid measure of adaptation in people diagnosed with IBD.

## Materials and methods

### Design

The study used a quantitative, cross sectional design.

### Sample

Participants were recruited as part of an opportunity sample from social networking sites, predominantly IBD support groups and forums on Twitter and Facebook, as well as dedicated IBD online support website forums. Participants were required to be at least 18 years of age, capable of providing informed consent, diagnosed with either indeterminate colitis, UC or CD and could be of any nationality providing they understood English. Participants described their gender, age, highest level of education, ethnicity and IBD diagnosis. In total 388 participants began the study and of these 309 completed the study giving a completion rate of 80%. Of the 309 completers, seven were diagnosed with indeterminate colitis, 83 with UC and 217 with CD. Two participants were excluded from the analysis as their diagnosis was described as IBS. Of the remaining 304 participants, 51 were male (16.8%) and 253 were female (83.2%) and the mean age was 34.23 years for the sample (range = 18–68 years). The most common level of qualification for the sample was undergraduate degree (*n* = 122 participants) and the most common ethnicity was White British (*n* = 151).

### Procedure

The study was advertised on social networking sites and support groups providing brief details concerning the aims of the study, a contact for those requiring further details and a hyperlink to the Qualtrics study page wherein the full information sheet, consent form and online adaptation questionnaire were contained. Ethical approval was obtained from the University of Surrey Ethics Committee (ref: EC/2014/157/FAHS) and declared to all patients prior to study commencement.

### Measures

In addition to the new Adaptation to IBD scale, participants also completed the Brief Illness Perception Questionnaire (Brief IPQ; Broadbent et al., 2006) and the Inflammatory Bowel Disease Questionnaire (IBDQ) (Guyatt et al., 1989) in order to assess convergent validity of the scale. The Brief IPQ assesses sense making in terms of representations relating to consequences, timeline, personal control, treatment control, identity, concern, coherence and emotion. This measure has been shown to have good test-retest reliability and convergent validity with relevant measures and has been used in a large variety of languages and countries (Broadbent et al., 2015). The IBDQ meanwhile assesses IBD-related quality of life as it pertains to bowel symptoms, systemic symptoms, emotional function and social function. This measure has also shown good convergent validity and internal reliability (Han et al., 1998) and is the most widely used measure of quality of life in IBD research (Fletcher et al., 2021).

#### Developing and validating the new measure of adaption to IBD (A-IBD)

The process of developing the new measure will be described through each step in turn and involved the stages of conceptualisation, operationalisation and assessments of the scale’s psychometric properties followed by convergent validity assessment with the Brief IPQ & IBDQ.

#### Conceptualisation

The previous qualitative study by Matini and Ogden (2015) identified three core themes from the experiences of those adapting to IBD. These were ‘making sense of the illness’, ‘impact of IBD’ and ‘feelings of IBD’. These themes find close parallels to models of sense making in chronic illness (Leventhal et al., 1980; Taylor, 1983). It was argued, however, that transcending these themes was the key theme of ‘uncertainty’ which was managed through a process of striving to find a new normal. Central to this process was the balance between the identity as a person (without the illness) and the identity as a patient (with the illness). Furthermore it was argued that this balance was achieved via a degree of acceptance and expectations of a positive future. In line with this, the present study conceptualised adaptation to IBD in terms of four constructs: identity as a person; identity as a patient; acceptance; expectations.

#### Operationalisation

The four constructs outlined above were each operationalised using 10 items derived from the qualitative study (Matini and Ogden, 2015). Examples are as follows:

*Identity as person:* “I exert myself just as much as I used to before my diagnosis”; “I go out and socialise regardless of any symptoms I may be experiencing”; “I try and live a normal life like everyone else”; “I must not let my illness get the better of me".

*Identity as a patient:* “I fear that my illness will stop me achieving my goals in life”; “If I exercise then my symptoms will return”; “I’m afraid to go out with friends in case I need to go to the toilet”; “Life will never be the same again".

*Acceptance:* “Some days I will not be as productive as I want to be”; “I am comfortable discussing my symptoms with my family and close friends”; “I get angry or upset when I experience symptoms”; “I feel like my illness is a burden".

*Expectations:* “I will be cured from my illness 1 day”; “I will live the life I had before being diagnosed”; “I will achieve everything I always wanted to in life”; “If my symptoms are under control then they will not return".

This created the 40 items included in the initial questionnaire which were each rated on a 5-point Likert scale ranging from ‘strongly disagree’ (1) to ‘strongly agree’ (5). A 5-point Likert scale was chosen as it has been suggested that scales of this length are less confusing for patients than lengthier scales and thus increase response rates (Taherdoost, 2019). A 5-point Likert scale also allows for the inclusion of a midpoint response option (‘neither agree nor disagree’) so that responders are permitted to express their true neutral or indifferent opinion if necessary (Chyung et al., 2017). 40 initial items were included to enable items to be excluded throughout the process of questionnaire development.

#### Assessment of the scale’s psychometric properties

The psychometric properties of the scale were assessed and refined using: i) data screening; ii) the principal axis factoring method of factor rotation to reduce the items into a factor structure that was both statistically and semantically coherent; iii) Cronbach’s alpha to assess internal consistency (reliability).

## Results

### Data screening

Using a range of criteria based upon the ratio between sample size and number of items (Comrey, 1973; Field, 2005; Kass and Tinsley, 1979) the sample of the current study (*n* = 304) was deemed appropriate for a factor analysis of 40 items. Furthermore, the Kaiser-Meyer-Olkin (KMO) statistic was 0.881 indicating that the sampling adequacy of the current data set could be evaluated as ‘meritorious’ (Kaiser, 1974). To explore inter correlation between variables a correlation matrix was computed for all 40 items. From this, Bartlett’s test of sphericity statistic was highly significant (*p* < 0.001) indicating that the data was factorizable. However, the correlation matrix was further assessed to investigate whether any one variable failed to correlate with at least one other variable at a value of less than 0.3 (Field, 2005). As such, four items (“I worry that when I go out with my friends I may suddenly need the toilet”, “I spend time researching medications or other treatment options”, “I am comfortable discussing my symptoms with my family and close friends” and “Surgery will completely turn things around for me”) were excluded from the analysis. Finally, the degree of ‘multicollinearity’, a statistical concept where several items in a model are highly correlated, was assessed using the determinant statistic (Field, 2005). The determinant was 0.00,000,105 which is less than the desired value. However, no two items were correlated above r = 0.8 suggesting that factor analysis was an acceptable approach.

### Principal axis factoring

Principal axis factoring was chosen as this was an exploratory study. Despite the items being derived from the results of Matini and Ogden’s (2015) qualitative study, these items theoretically informed but did not dictate the analysis. The thematic structure of this prior qualitative study was thus not aiming to be confirmed by the current study.

The principal axis factoring exploratory analysis method takes into account both the latent variables that contribute to the underlying factor structure and the difference between shared and unique variance and was also chosen as the data was largely not normally distributed (Fabrigar et al., 1999). The oblique rotation method was chosen for the current analysis to aid interpretability as well as allowing for the underlying factors to correlate with one another (Field, 2005). The data was then run through a series of factor analyses as follows. Criteria for inclusion or any given item were: a cut off of factor loading >0.4, a communality statistic of >0.3 and no cross loading.

*Solution 1:* Initial principal axis factoring revealed 11 factors that had eigenvalues greater than 1.0 and which explained 21.4%, 4.9%, 4.3%, 4.0%, 2.4%, 1.8%, 1.6%, 1.5%, 1.3%, 1.2% and 1.1% of the total variance accounting for 45.4% of the total variance explained. However, the scree plot indicated a four factor solution (Cattell, 1966).

*Solution 2:* A forced four factor solution was run in line with the scree plot. The resulting factors explained 22.7%, 4.8%, 4.3% and 3.4% of the total variance, accounting for a total of 35.0% of the total variance. Factors 1 and 2 both consisted of eight items, factor 3 consisted of five items and factor 4 consisted of three items. The scree plot still showed a four factor solution. However, due to loading on more than one factor or having a communality statistic >0.3, 12 items were removed leaving 28 items.

*Solution 3:* A forced four factor solution was re run with these 28 items. The scree plot again supported a four factor solution with the four factors now explaining 23.6%, 6.6%, 5.3% and 4.0% of the total variance, accounting for an improved total of 39.4% of the total variance. A further two items were removed due to their low communality statistic (0.152) and high cross-loading on another factor. This left 26 items.

*Solution 4:* The next analysis was run on these 26 items. The factors this time explained 23.8%, 6.7%, 5.4% and 4.2% of the total variance, accounting for an improved total of 40.2% of the total variance explained. From this analysis a final eight items were removed due to low communality and high cross loading. This left the final solution of a scale with 18 items.

*The final solution:* The final solution with 18 items consisted of four factors with no cross loading and with all factor loadings>0.4. They were labelled as follows: Factor 1 was labelled ‘Patient identity’ and consisted of six items, (e.g. “I fear that my illness will stop me achieving my goals in life”; “I feel like my illness is a burden”, “Life will never be the same as it was before my diagnosis”); factor 2 consisted of five items and was labelled ‘Person identity’ (e.g. “I exert myself just as much as I used to do before my diagnosis”; “I try and live a normal life like everyone else”; “I still keep up my hobbies and interests that I had before my diagnosis); factor 3 consisted of four items and was labelled ‘Expectations’ (e.g. “I expect my doctor will eventually find a medication that solves my problems”; “I expect things will only get easier with time”; “I believe my fatigue will sort itself out eventually”) and factor 4 consisted of three items and was labelled ‘Acceptance’ (e.g. “I accept that I have a chronic illness which has no cure”; “I am accepting of the possibility of future flare ups of my symptoms”; “I just have to keep moving forward with my life”). These four factors were corroborated by the scree plot, with the factors accounting for 22.8%, 8.0%, 5.5% and 4.8% with the total explained variance being 41.1%. The KMO statistic for this final solution was ‘meritorious’ at 0.851 and the determinant value now indicated no concern for multicollinearity at 0.009. In addition, Bartlett’s test of sphericity was once again highly significant at *p* < 0.001. The factor loadings for each factor and its items are shown in Table 1.

**Table 1.** Factor loadings for 18-item adaptation questionnaire (*n* = 304).

*Rotated factor loadings*				
Item	1. Patient identity	2. Person identity	3. Acceptance	4. Expectations
I fear that my illness will stop me achieving my goals in life	**−0.62**	−0.16	0.04	0.05
I feel like my illness is a burden	**−0.63**	−0.16	0.11	0.00
Life will never be the same as it was before my diagnosis	**−0.58**	−0.11	−0.14	**−0.12**
I get angry or upset when I experience symptoms	**−0.59**	0.13	0.11	0.00
I wonder about how my health will be in the future	**−0.59**	0.02	−0.12	0.03
There is nothing I can do to make myself feel better	**−0.52**	−0.03	0.06	−0.17
I exert myself just as much as I used to do before my diagnosis	0.13	**0.67**	0.14	−0.06
I try and live a normal life like everyone else	0.00	**0.64**	−0.02	0.01
I still keep up with my hobbies and interests that I had before my diagnosis	0.17	**0.63**	−0.02	−0.00
I ignore any fatigue I may be experiencing and try to function as normal	**−0.14**	**0.62**	−0.04	0.02
I go out and socialise regardless of any symptoms I may be experiencing	0.07	**0.49**	−0.02	0.13
I accept that I have a chronic illness which has no cure	−0.08	−0.07	**−0.68**	0.00
I am accepting of the possibility of future flare ups of my symptoms	0.09	−0.06	**−0.61**	−0.12
I just have to keep moving forward with my life	0.04	0.26	**−0.41**	0.08
I expect my doctor will eventually find a medication that solves my problems	−0.01	**−0.08**	−0.11	**0.68**
I expect things will only get easier with time	0.19	0.07	−0.02	**0.59**
I believe my fatigue will sort itself out eventually	0.09	0.10	0.05	**0.56**
I will be cured from my illness 1 day	**−0.11**	0.00	0.16	**0.54**

Factor loadings>0.4 are highlighted in bold.

### Reliability assessment

Cronbach’s alphas were calculated for each subscale to assess their internal reliability. Patient identity (6 items) was 0.8; person identity (5 items) was 0.8; expectations (4 items) was 0.7 and acceptance (3 items) was 0.6. All factors were found to have relatively good levels of internal reliability. Furthermore, none of these alphas could be improved by deleting items and no items from any of the factors had a corrected item-total correlation of less than 0.41, suggesting all items correlated well with their respective factors overall.

## Convergent validation of the new scale against existing measures

In order to provide support for the distinctness of the prospective Adaptation to IBD scale (A-IBD) the sub scales were computed, descriptive statistics were run and these scores were correlated with the subscales of the Brief IPQ and the IBDQ. Descriptive statistics indicated that the mean ‘patient identity’ score was 3.67 (SD = 0.71), the mean ‘person identity’ score was 3.10 (SD = 0.82), the mean ‘acceptance’ score was 4.3 (SD = 0.54) and the mean 'expectations' score was 2.46 (SD = 0.76). The correlation matrix is shown in Table 2.

**Table 2.** Pearson correlations between the A-IBD, the Brief IBQ & the IBDQ.

	Brief IPQ								IBDQ			
**A-IBD**	Consequences	Timeline	Personal Control	Treatment Control	Identity	Concern	Coherence	Emotional representation	Bowel symptoms	Emotional function	Systemic symptoms	Social function
Patient identity	0.46**	−0.15*	0.34**	0.28**	−0.44**	−0.33**	−0.06	−0.39**	−0.22**	−0.40**	−0.33**	−0.31**
Person identity	−0.48**	−0.15*	0.34**	0.28**	−0.44**	−0.34**	−0.06	−0.39**	0.27**	0.36**	0.37**	0.44**
Acceptance	−0.07	0.19**	0.18**	0.12*	−0.08	−0.09	0.33**	−0.10	−0.00	0.07	−0.02	0.03
Expectations	−0.25**	−0.30**	0.26**	0.23**	−0.28**	−0.21**	−0.12*	−0.29**	0.18**	0.26**	0.27**	0.27**

*p < 0.05; **p < 0.01.

The results showed that ‘Patient identity’ was significantly positively correlated with the consequences, personal control and treatment control subscales of the Brief IPQ and negatively correlated with timeline, identity and concern**,** while being negatively correlated with all subscales of the IBDQ. ‘Person identity’ was positively correlated with personal control and treatment control of the Brief IPQ but negatively correlated with most other subscales, while being positively correlated with all subscales of the IBDQ. The scale reflecting ‘Acceptance’ was positively correlated with timeline, personal control, treatment control and coherence but unrelated to consequences, identity, concern and emotion of the Brief IPQ, while being unrelated to any subscale of the IBDQ. Finally, ‘Expectations’ was positively correlated with personal control and treatment control of the Brief IPQ but negatively correlated with consequences, timeline, identity, concern, coherence and emotion. The subscales of the A-IBD therefore show some relationships with sense making as measured by the Brief IPQ and quality of life as measured by the IBDQ. However, the highest correlation coefficient was −0.48 (‘person identity’ of the A-IBD and ‘consequences' of the Brief IPQ) which was only moderate in strength. These results indicate that although this new measure shares some of the variance with existing scales, indicating convergent validity as desired, it is also making a novel contribution to the concept of adaptation.

## Discussion

Research indicates that IBD impacts upon patients’ daily lives in a multitude of ways and that adaptation to this condition is imperative if people are to manage their condition effectively. To date, however, although much has been written about adaptation to chronic illnesses in general, and in particular IBD, exactly what this construct encapsulates and how it should be measured remains unclear. The aim of the present study was therefore to provide a clear conceptualisation of adaptation in the context of IBD as a means to operationalise this construct and develop a reliable measure. Adaptation in this study was initially conceptualised according to the qualitative findings of Matini and Ogden (2015) - reflecting a balance between the identity as a person and that of a patient resulting in a degree of equilibrium which was achieved through a degree of acceptance and positive expectations about the future. Subsequently, this conceptualisation was operationalised using 40 items based upon the prior qualitative work to reflect this definition. Psychometric testing in the form of an exploratory factor analysis then produced an 18-item solution with four factors reflecting patient identity, person identity, acceptance and expectations which were shown to have a robust factor structure and good internal consistency as measured by the Cronbach’s alphas and corrected item-total correlations of the factors. The measure was then further assessed for convergent validity through correlation analyses with an existing measure of sense making (the Brief IPQ) and quality of life (the IBDQ). This indicated a mixture of weak to moderate associations suggesting that whilst adaptation overlaps to a degree with sense making in IBD and IBD relate quality of life, it is not coterminous and therefore measuring something distinct and worthwhile.

Some considerations must be made however when interpreting the findings of the current study. It should firstly be noted that there was no way of ascertaining whether the participants who completed the study had a concrete, medical diagnosis of IBD, as opposed to perhaps IBS or a self-diagnosis of IBD, despite the inclusion criteria being stated prior to participation. Importantly, it should also be taken into account that due to the exploratory nature of the factor analysis carried out, the subsequent 18-item scale must still be considered as in development and requiring further psychometric testing before being considered entirely statistically valid, reliable and appropriate for use by other researchers or practitioners. Future research is therefore needed to further develop the psychometric robustness of the scale and confirm its structure by administering it to a different patient sample and carrying out a confirmatory factor analysis on this new data. In particular, the ‘acceptance’ subscale may need more statistical support for its inclusion in an eventual final model following a confirmatory factor analysis due to it having the fewest factors loadings and lowest Cronbach’s alpha. The subscale was retained as part of the four-factor solution for the time being as the current study sought to balance psychometric rigour with as accurate a reflection of the patient experience as possible. A further potential limitation that must be acknowledged is that there is a discrepancy between the development of a static measure of adaptation and the notion put forward of adaptation being a relational, iterative process. This has implications for the measure in terms of its translation into practice. It is likely therefore that any eventual inclusion of measures relating to adaptation, such as the A-IBD, need to be repeated with patients to assess any fluctuations in adaptation over time which raises potential issues of pragmatism.

However, the potential benefits of the study likely outweigh these limitations. The scale could, for example, better the understanding of the notion of adaptation in chronic illness and subsequently promote the development of similar measures for other chronic conditions. In doing so, it could potentially complement the more commonly utilised patient reported outcome measures such as quality of life. Ultimately, measures such as the A-IBD have the potential to be utilised by psychologists within healthcare teams to quickly measure adaptation longitudinally and better optimise patients’ degree of adaptation to their illness over time. This in turn has the potential to improve their psychological as well as functional wellbeing and subsequently ideally also their disease burden. The potential benefits of this for health care teams are significant in the form of potentially less clinic footfall, for example. This is indeed of particular relevance in the current COVID-19 pandemic era, and glaringly so in IBD care (Kennedy et al., 2020) where clinical resources, such as outpatient clinic capacity and the ability to respond to IBD helpline activity, have been stretched.
